# Moderating Effects of Exercise Duration and Intensity in Neuromuscular vs. Endurance Exercise Interventions for the Treatment of Depression: A Meta-Analytical Review

**DOI:** 10.3389/fpsyt.2018.00305

**Published:** 2018-07-19

**Authors:** Lukas Nebiker, Eric Lichtenstein, Alice Minghetti, Lukas Zahner, Markus Gerber, Oliver Faude, Lars Donath

**Affiliations:** ^1^Department of Sport, Exercise and Health, University of Basel, Basel, Switzerland; ^2^Department of Intervention Research in Exercise Training German Sport University Cologne, Köln, Germany

**Keywords:** endurance, exercise, duration, intensity, major depressive disorder, RCT, strength, training

## Abstract

**Background:** Exercise training is a beneficial treatment strategy for depression. Previous meta-analytical reviews mainly examined the effect of aerobic exercise on depressive symptoms neglecting comparisons with neuromuscular training and meta-regression considering relevant exercise training prescriptors such as exercise duration, intensity, number of exercise sessions (volume) and frequency.

**Methods:** A structured literature search was conducted in biomedical and psychological databases and study selection was conducted following the PICOS approach. (Randomized) controlled trials that compared supervised neuromuscular or endurance exercise interventions with an inactive control group (CON) in clinically depressed in- or out-patients over 18 years were included. Eligibility and study quality were evaluated by two independent researchers. Standardized mean differences (SMD) for the reduction of depressive symptoms, measured with different evaluation scales (e.g., BDI, HAM-D, PHQ-9, HRSD, MADRS, GDS) were calculated with the adjusted Hedges'g equation as main outcome for the comparison of endurance and neuromuscular exercise interventions vs. CON. Statistical analyses were conducted using a random effects inverse-variance model. Multivariate meta-regression analysis was performed in order to examine the modulating effects of exercise training prescriptors.

**Results:** Twenty seven trials with 1,452 clinically depressed adults were included. 20 out of 27 included trials reached a PEDro score of at least 6, representing high-quality. Irrespective of the exercise mode and study quality, large effects in favor of exercise compared to the control condition were found. Compared to CON, sensitivity analyses revealed a moderate to large effect in favor of endurance exercise [SMD: −0.79 (90% CI: −1.10, −0.48); *p* < 0.00001, *I*^2^ = 84%] and a large effect size in favor of neuromuscular exercise [SMD: −1.14 (90 CI: −1.50, −0.78); *p* < 0.00001, *I*^2^ = 80%]. These effects decreased to moderate for endurance and remained large for neuromuscular trials when considering studies of high quality, indicating a significant difference (*p* = 0.04). Multivariate meta- regression revealed that exercise duration in endurance trials and exercise intensity in neuromuscular trials had a significantly moderating effect.

**Conclusions:** Strong neuromuscular exercise interventions can be slightly more effective than endurance exercise interventions. Interestingly, exercise duration and exercise intensity moderated the effect size meaningfully. This result might be used on exercise in depression to increase efficacy.

## Introduction

Depression is considered a leading cause of disability worldwide and a major contributor to the overall global burden of disease (1). According to the WHO (1), several effective treatments for depression exist but less than half of the affected people receive such treatments. According to Ebmeier et al. (2) and Halliwell et al. (3), merely 18–25% of the depressed patients receive an adequate treatment with antidepressant medication and psychotherapy.

Moreover, the treatment with antidepressant medication is accompanied with poor compliance (4) and has been reported to cause several unintended side effects like withdrawal symptom (2), nausea, insomnia, anxiety (5), weight gain (6), or sexual dysfunction (7). Therefore, further evidence-based alternative or complementary treatment approaches for depressive disorders are needed.

The WHO (8) and the NICE (9) guidelines recommend physical exercise as a standard complementary treatment option for depression. Exercise as a complementary treatment option provides various benefits such as decreased blood pressure (10), weight reduction (11), increased oxygen uptake (12, 13) while negligible side effects are known (11).

The beneficial effect of physical exercise in the treatment of depression has previously been examined in several meta-analyses. Due to a large heterogeneity of included studies in terms of study quality, diagnosis of depression, mode of exercise, included subjects and duration, the effect sizes given as standardized mean difference (SMD) range between small effects in favor of exercise [−0.34 (14) to −0.40 (15)], moderate effects [−0.77 (16) to −0.72 (17)] or significant large effects [−0.80 (10), −0.82 (18), −1.1 (19), −1.1 (20), and −1.39 (21)]. Interestingly, meta-analyses that included only methodological strong trials revealed lower effect sizes. For instance, Krogh et al. (22) and Mead et al. (18) reported SMDs of −0.19 and −0.42, respectively. Another example is the meta-analysis of Lawlor and Hopker (19), which resulted in an effect size of −0.69 after removing 41% of all studies due to poor methodological quality (23).

Even though the antidepressant effects of exercise for the treatment of depression are well-understood, the moderating effect of training prescriptors (e.g., exercise frequency, intensity, duration of sessions, number of sessions) and the difference between neuromuscular vs. endurance training remains elusive to date. A differentiation between neuromuscular and endurance exercise seems beneficial as patients do have different exercise preferences and both exercise modes cause different adaptations on behavioral and molecular level (24). Against this background, the purpose of the present systematic review and meta-analysis with meta-regression is to (a) update the effects of physical exercise in the treatment of depression following precise inclusion criteria and sound methodological quality, (2) examine the effects of endurance exercise interventions and neuromuscular exercise interventions on depression, and (3) execute a meta-regression analyses including the different training parameters, which could influence the effect sizes of exercise on depression.

## Methods

### Search strategy

The present meta-analysis was performed along the PRISMA guidelines (25). Biomedical and psychological databases (PubMed, SPORTDiscus, CINAHL, Cochrane Library, ProQuest Database) were screened from the 17th of February to the 28th of November 2017. Similar key words and Boolean conjunctions (OR/AND) were used as in the meta-analysis of Schuch et al. (26): [(exercis^*^ OR aerobic^*^ OR running^*^ OR jogging^*^ OR walk^*^ OR hiking OR swim^*^ OR aquatic^*^ OR cycling OR bicycl^*^ OR strength^*^ OR flexibility AND activit^*^ OR fitness OR train^*^ OR “physical medicine” OR resistance OR lift^*^) AND [depress^*^ OR dysthymia]].

**Figure 1:**
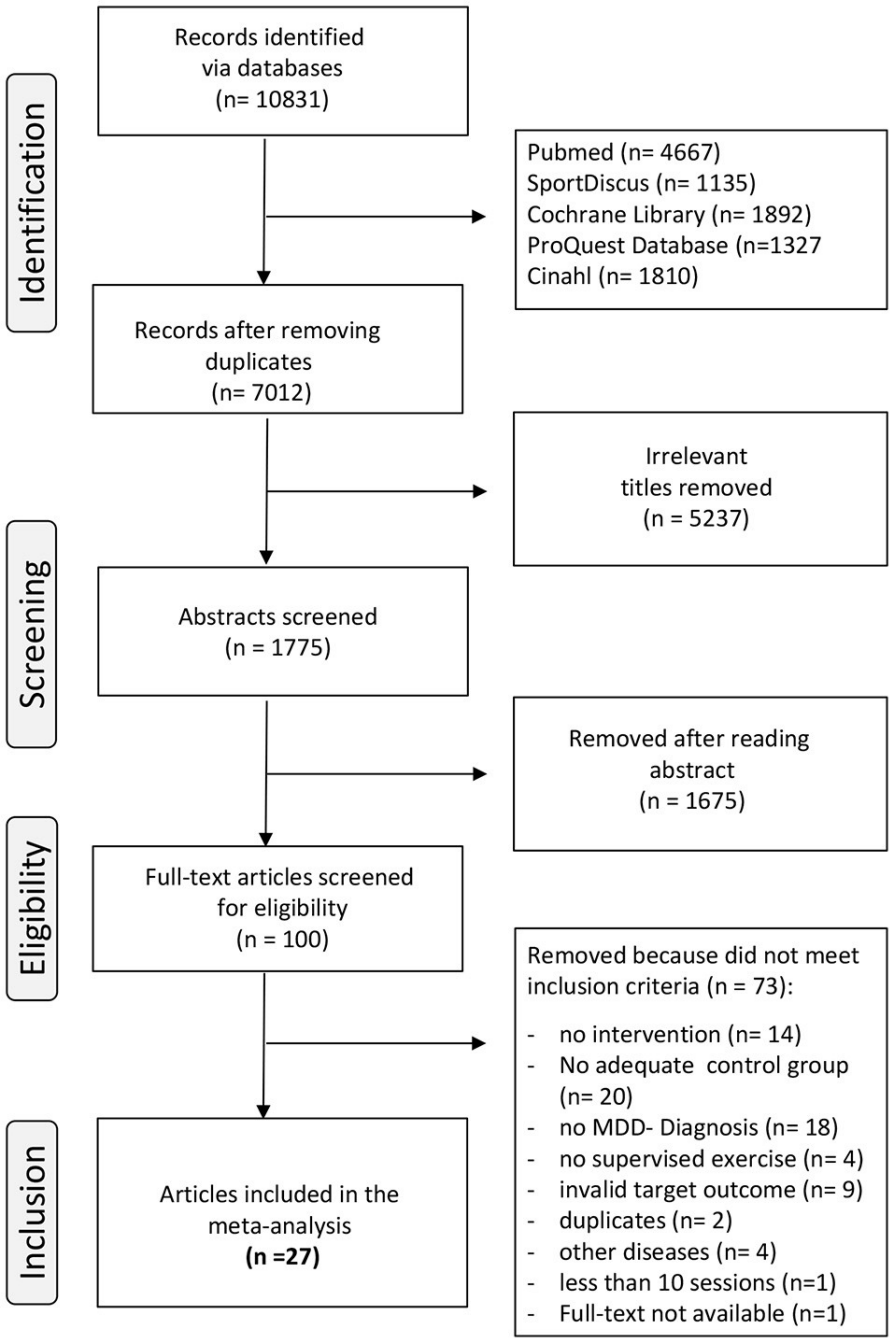
Flow chart of the different phases of study screening and selection.

In addition, recent reviews and cited articles about exercise and depression were screened and potentially eligible articles were added to the library. Duplicates were identified and excluded. The remaining studies were gradually screened using the titles, abstracts and full-texts of the potentially eligible articles (Figure 1). The final decision for inclusion or exclusion was made by two independent authors (LN, LD) based on the inclusion criteria.

### Inclusion criteria

Eligible studies had to meet the following inclusion criteria based on the PICOS approach (27) for population (P), intervention (I), comparators (C), main outcome (O), and study design (S):

- (P) Participants had to be ≥ 18 years of age and were either diagnosed using the Diagnostic and Statistical Manual of Mental Disorders, Fourth Edition (DSM-IV) criteria for MDD, a diagnosis of MDD with the International Classification of Diseases (ICD-10), 10th revision, a diagnosis of MDD using the Research Diagnostic criteria (RDC), a diagnosis for depression using the Structured Clinical Interview for Depression (SCID), the 21-item Beck Depression Inventory II (BDI-II), the Geriatric Depression Scale (GDS), or the 9-item Patient Health Questionnaire (PHQ-9).
- (I) The (randomized) controlled trials should investigate exercise in the sense that improvement or maintenance of at least one component of physical fitness was the objective. Physical exercise sessions had to be supervised by coaches, medical students or similar experts. The exercise interventions could either be aerobic exercises, strength exercises, functional exercise training, yoga or tai chi. The participants completed at least 10 physical exercise sessions.
- (C) There must be a control group (CON), which did not follow a physical exercise intervention like aerobic exercise, strength exercise, yoga exercise, stretching, or relaxation exercise. The control group can either be a control group with behavioral therapy, light therapy, medication therapy, a placebo-group, or an inactive control group.
- (O) At least one outcome for the depression score had to be evaluated in the study. That could either be the Beck Depression Score (BDI) or BDI-II, Hamilton Rating Scale for Depression (HAM-D, HRSD, HDRS), Montgomery-Åsberg Depression Rating Scale (MADRS), Center for Epidemiological Studies Depression Scale (CES-D), the Geriatric Depression Scale (GDS), or the 9-item Patient Health Questionnaire (PHQ-9). Depressive symptoms must be measured or reported pre- and post-intervention.
- (S) Studies had to be (randomized) controlled interventions with pre- and post-testing and an intervention duration of at least 10 days and 10 sessions, respectively.

### Exclusion criteria

Studies were excluded when they met one of the following criteria: (1) children or adolescents <18 years old. (2) Participants without a diagnosis of depression. (3) Participants with chronic illnesses or further diseases apart from MDD (e.g., diabetes). (4) Inappropriate physical exercise interventions or interventions without physical exercise (e.g., mindfulness-based stress reduction therapy). (5) Exercise interventions with non- supervised sessions. (6) Invalid target outcomes (see inclusion criteria). (6) No adequate control condition or control group.

### Assessment of methodological quality of the studies

The methodological quality of all eligible trials was assessed using the PEDro (Physiotherapy Evidence Database) scale. The PEDro scale contains 11 dichotomous (yes or no) items, in which the criteria 2–9 rate randomization and internal validity and the criteria 10–11 rate the presence of statistical replicable results. Criterion 1 relates to the external validity but will not be used to calculate the PEDro score.

Studies were rated independently by two non-blinded reviewers (LN and EL) and they needed to obtain consensus on every item. Discordant study ratings were discussed point to point by the two reviewers (LN and EL) to come to a decision. To represent a high-quality study, the PEDro score had to be ≥6 on the scale from 0 to 10.

### Outcome

The intervention was the mean change in depressive symptoms from baseline to post-intervention measures in the exercise groups compared to the control groups. Stratification to sex, medication and severity was not doable due to the small resulting subgrouping and overall power of the calculations. The standardized outcome was calculated as the standardized mean differences (SMD) and presented as means together with 90% confidence intervals (CI). If included studies contain more than one outcome measure meeting our criteria (mean change pre-and post in depressive symptoms), we prioritized the common scales BDI/BDI-II or HAMD to further minimize heterogeneity of our findings [e.g., (28)]. In studies reporting more than one exercise group (differing in exercise intensity or type of exercise), all exercise groups were included in the meta-analysis and allocated either to endurance exercise intervention or neuromuscular exercise intervention [e.g., (29)]. The latter category included traditional strength or resistance training as well as Yoga or Tai-Chi based exercise regimen. Those approaches were initially analyzed independently and further pooled to “neuromuscular training” once they did not reveal significantly different effect sizes.

### Data extraction and categorization

The following data were extracted by two researchers (LN and EL) and transferred to an excel spread sheet: sample (number of participants in the intervention group and in the control group), exercise (type of exercise, number of sessions, duration of one session, weekly frequency, intensity of the sessions), outcome [pre- and post-test means and standard deviations (SD)]. Exercise intensity was described in the studies in several ways. For the sake of comparability, the different measured values (i.e., maximum oxygen uptake, heart rate reserve or Borg Scale) were categorized in the following intensity groups to make the comparison of underlying exercise intensities more suitable: low intensity (40% of maximal heart rate), low to moderate intensity (50%), moderate intensity (60%), moderate to high intensity (70%), and high intensity (80%). The exercise intensity in trials with Tai Chi or Yoga exercise interventions was not described thus we assumed low to moderate intensity.

Further relevant study information concerning reference (author and date of publication), study- design, number of participants, mean age, interventional characteristics (experimental and control group) as well as training characteristics and outcome measures were additionally described in Table 1 (study overview for neuromuscular exercise interventions) and Table 2 (study overview for endurance exercise interventions). All intervention trials that focused on aerobic or endurance exercise with a more pronounced cardiovascular stimulus were categorized as “endurance” and all exercise that entail coordination exercises or strength elements were categorized as “neuromuscular.”

**Table 1:** Study overview of neuromuscular exercise interventions.

	**References**	**Study design**	**Sample: Population, Sample size (*n*), Age (years)**	**Groups**	**Intervention**	**Training characteristics**	**Outcome measures**
1	(30)	Randomized, controlled trial	Sedentary women with a BDI-II score over 14, aged 18–50 years, *n* = 26; INT: 33.08 ± 9.11 year CON: 32.38 ± 8.27 year	INT (*n* = 13) CON (*n* = 13)	(a) INT, croup based yoga sessions with 5 min of pranayama (breathing exercise), 5 min warm-up, 40 min of asana (yoga poses) practice and 10 min of savasana (meditation/relaxation). No intensity described, assumption to be low to moderate intensity (b) CON, instructed to maintain their usual level of physical activity during the study duration	12 weeks, 2 sessions/week (24 training sessions); each session lasted 60 min	BDI-II
2	(29)	Randomized, controlled trial	Women with diagnosed depression (Research Diagnostic criteria), aged 18–35 years *n* = 40; 28.52 ± 4.36 year	INT (*n* = 14) INT (*n* = 15) 3) CON (*n* = 11)	(a) INT, aerobic exercise prescription on the other over- view (b) INT, 5–10 min warm-up, a standard 10-station program with a heart rate below50–60% of maximum heart rate and a 5–10 min cool-down period (c) CON, wait- list control group	8 weeks, 4 sessions/week (32 training sessions); no general exercise duration	BDI and HRSD
3	(31)	Randomized, controlled trial	Pregnant women with diagnostic criteria for depression on SCID, <40 years old, *n* = 92; INT: 24.4 ± 4.7 year CON: 26.0 ± 5.6 year	INT (*n* = 46) CON (*n* = 46)	(a) INT, yoga/tai- chi group sessions with a duration of 20 min. No intensity described, assumption that Tai- Chi lessons have moderate intensity (b) CON, wait- list control group; no intervention	12 weeks, 1 session/week (12 training sessions); each session lasted 20 min	CES-D
4	(32)	Comparative, controlled trial with an open- labeled design	Out-patients, fulfilled DSM-IV criteria for MDD, *n* = 58; 33.65 y mean age	INT (*n* = 15), not in meta- analysis included INT (*n* = 27) CON (*n* = 16)	(a) INT, yoga only, not in the meta- analysis involved (b) INT, yoga lessons with a duration of 60 min per lesson consisting of yogasana and breathing procedures. No intensity described, assumption to be low to moderate intensity. Furthermore, they received antide-pressant medications with a dose defined by the treating psychiatrist	First 2 weeks daily train- ing, next 2 weeks weekly interval (16 training ses- sions); each session lasted 60 min	HRSD
5	(33)	Randomized, controlled trial	Older adults over 65 years, with a GDS- score > 5, *n* = 57; 76.53 ± 5.94 year	INT (*n* = 19) INT (*n* = 18), not in meta- analysis in- cluded CON (*n* = 20)	(a) INT, physical exercises including a warm-up, cardio- vascular exercises (walking with waving or clapping hands), muscle strength exercises (triceps brachii, biceps brachii, quadriceps femoris, iliopsoas) with a rated Borg Scale score between 12 and 14 and a cool-down (b) INT, cognitive behavioral therapy, not in meta-analysis included (c) CON, they receive no extra care	12 weeks, 3 sessions/week (36 training sessions); each session lasted 50 min	GDS-15
6	(34)	Randomized, controlled trial	Older adults over 60 years, fulfilled DSM- IV criteria for MDD, *n* = 73; INT: 69.1 ± 7.0 year CON: 72.0 ± 7.4 year	INT (*n* = 36) CON (*n* = 37)	(a) INT, 2 h of Tai Chi Chi; TCC employs “meditation through movement” No intensity described, assumption to be moderate intensity (b) Furthermore, they received 10 mg/day of escitalopram (c) CON, they received a Health Education Protocol. Furthermore, they received 10 mg/day of escitalopram	10 weeks, 1 session/week (10 training sessions); each session lasted 120 min	HRSD
7	(35)	Randomized, controlled trial	Outpatient with a GDS- score > 10, aged over 53 years, *n* = 86; INT: 63.7 year CON: 66.2 year	INT (*n* = 43) CON (*n* = 43)	(a) INT, exercise classes with predominantly weight- bearing exercise performed to music. There was a warm-up period of 5–10 min and a cool-down period at the end. No intensity described (b) CON, they received twice weekly health education talks for 10 weeks	10 weeks, 2 sessions/week (20 training sessions); each session lasted 45 min	HRSD and GDS
8	(36)	Randomized, naturalistic con- trolled trial	Women, aged 40–60 years, fulfilled DSM- IV criteria for MDD, *n* = 30; no mean age	INT (*n* = 10) CON (*n* = 20)	(a) INT, each session included a warm up (5 min), physiological strengthening with machines (50 min) and a cool-down (5 min). The exercise machines allowed different exercises for arms, legs and postural muscles and were changed every 4 min. There is no specific exercise intensity described. Further- more, they received pharmacological therapy (b) CON, they only tot pharmacological therapy	32 weeks, 2 ses- sions/week (64 training sessions); each session lasted 60 min	HAM-D
9	(37)	Randomized, controlled pilot trial	Aged over 18 years, diagnosis of MDD, *n* = 38; 43.4 ± 14.8 year	INT (*n* = 20) CON (*n* = 18)	(a) INT, 90- min practice sessions comprised of classical yoga breathing techniques, mindful body postures and final deep relaxation pose. No exercise intensity described, assumption that hatha yoga is low intensity exercise (b) CON, 90-min education modules on yoga history and philosophy	8 weeks, 2 sessions/week (16 training sessions); each session lasted 90 min	BDI
10	(38)	Randomized, controlled trial	Aged 60 years or older, fulfilled DSM- IV criteria for MDD, *n* = 32; INT: 70 ± 1.5 year CON: 72 ± 2.0 year	INT (*n* = 17) CON (*n* = 15)	(a) INT, a high intensity (80% of one repetition maximum) progressive resistance training for the large muscle groups with 3 sets of 8 repetitions on each machine. The exercises included chest press, lat pulldown, leg press, knee extension and knee flexion. (b) CON, control subjects engaged in a health education program of lectures and videos	10 weeks, 3 sessions/week (30 training sessions); each session lasted 50 min	BDI
11	(39)	Randomized, controlled trial	Aged 60–85 years, fulfilled DSM-IV criteria for MDD, *n* = 60; INT: 69 ± 5 year INT: 70 ± 7 year CON: 69 ± 7 year	INT (*n* = 20) INT (*n* = 20) CON (*n* = 20)	(c) INT, a high intensity (80% of one repetition maximum) progres- sive resistance training for the large muscle groups with 3 sets of 8 repetitions. Exercise machines included chest press, lat pulldown, leg press, knee extension and knee flexion (d) INT, same procedure as a) but with low- intensity (20% of one repetition maximum) (e) CON, received usual care	8 weeks, 3 sessions/week (24 training sessions); each session lasted 65 min	HRSD and GDS

**Table 2:** Study overview of endurance exercise interventions.

	**References**	**Study design**	**Sample: Population, Sample size (n), Age (years)**	**Groups**	**Intervention**	**Training characteristics**	**Outcome measures**
1	(40)	Randomized, controlled trial, 3 arms	Older adults ≥50 years, fulfilled DSM- IV criteria for MDD, *n* = 156; 57 ± 6.5 y	INT (*n* = 55) CON (*n* = 48) Exercise only group not in- volved in meta- analysis	(a) INT, aerobic exercise sessions with a 10-min warm- up, 30 min walking or jogging with 70–85% of HRR and 5 min cool-down exercises; furthermore, patients received sertraline dosage of 50 mg up to 200 mg daily (b) CON, received only sertraline dosage of 50 mg up to 200 mg daily	16 weeks, 3 ses- sions/week (48 training sessions); each session lasted 45 min	HAMD-D and BDI
2	(41)	Randomized, parallel group, pla- cebo-con- trolled trial	Outpatients, fulfilled DSM-IV criteria for MDD, aged over 40 years, *n* = 202; 52 ± 8 y	INT (*n* = 51) CON (*n* = 49) Medication group and home- based exercise group not in- volved in meta- analysis	(a) INT, aerobic exercise sessions with a 10-min warm- up, 30 min walking or jogging with 70–85% of HRR and 5 min cool-down exercises. (b) CON, received placebo- pills with a dosage of 50 mg up to 200 mg daily	16 weeks, 3 ses- sions/week (48 training sessions); each session lasted 45 min	HAM-D
3	(42)	Randomized, controlled trial, 2 arms	Women aged 18–65 years, with ICD-10 diagnosis for depres- sion, *n* = 19; INT: 52.78 ± 7.66 y CON: 47.80 ± 15.05 y	INT (*n* = 9) CON (*n* = 10)	(a) INT, aerobic exercise sessions with a 10- min warm up, 30 min of aerobics with 65–75% of maximal heart rate and 5 min cool- down (b) CON, continued their usual pharmacological therapy but without any additional exercise to their habitual ac- tivities	16 weeks, 3 session/week (48 training sessions); each session lasted 45–50 min	BDI-II
4	(43)	Not random- ized, con- trolled trial	Women, aged 20–64 years, with ICD-10 diagnosis for depres- sion, *n* = 82; INT: 33.1 ± 5.4 y CON: 31.7 ± 6.8 y	INT (*n* = 41) CON (*n* = 41)	(a) INT, progressive program of cardiovascular exercise with a warm-up, low- impact aerobics gymnastics, fun dance and walking and a cool- down. Exercise inten- sity is not described but we assumed that low-impact exercises have moderate exercise intensity. Further- more, the patients received 20 mg of Fluoxetine (b) CON, only received 20 mg Fluoxetine	8 weeks, 3 sessions/ week (24 training sessions); session duration increased from 45 to 60 min	BDI
5	(44)	Single-site, three- armed, randomized controlled trial	Adults, aged 18–65 years, fulfilled DSM- IV criteria for MDD, *n* = 62; INT: 44.7 ± 12.5 y CON: 46.3 ± 13.9 y	INT (*n* = 22) INT (*n* = 20), not in meta-analysis included CON (*n* = 20)	(a) INT, aerobic exercise sessions with a warm- up phase of 5–10 min, 45 min of interval training (inten- sity 16–17 on the Borg Scale) and 5 min cool- down phase with stretching exercise (b) INT, Basic Body Awareness Therapy (BBAT) inter- vention, not involved in meta- analysis (c) CON, participants only receive once advice and moti-	8 weeks, 2 sessions/ week (16 training sessions); each session lasted 60 min	MADRS
6	(45)	Pragmatic, randomized, controlled trial	Adults, slightly over- weight, aged 18–65 years, with ICD-10 diagnosis of depression, *n* = 46; 47.87 ± 10.47	INT (*n* = 30) CON (*n* = 16)	(a) INT, sessions with 10–15 min warm-up, 30–40 min walk- ing/running (average Borg Scale Score 11.6) and 10–15 min cool-down. Participants could self- select the exercise intensity. (b) CON, no intervention	8 weeks, 3 ses- sions/week (24 training sessions); each session lasted 60 min	BDI-II
7	(29)	Randomized, controlled trial	Women with diagnosed de- pression (Research Diag- nostic criteria), aged 18–35 years, *n* = 40; 28.52 ± 4.36	INT (*n* = 14) INT (*n* = 15) CON (*n* = 11)	(a) INT, 5–10 min warm-up, walking or running with 80% of maximal work capacity on an indoor track, 5–10 min cool- down (b) INT, weight lift condition prescription CON, wait-list control group	8 weeks, 4 ses- sions/week (32 training sessions); no general exercise duration	HRSD and BDI
8	(46)	Randomized, controlled, quasi- experi- mental trial	Female students, diagnosed with MDD, aged 18–25 years, *n* = 20; No mean ae	INT (*n* = 10) CON (*n* = 10)	(a) INT, 10 min warm-up, three sets of six min running with moderate intensity (60–65% of maximal heart rate) and 3 min relax- ing between the sets. Each week, 1 min had been added to the run- ning time of each set (b) CON, asked to pursue their normal life and do not have any phys- ical activity during the intervention period	8 weeks, 3 ses- sions/week (24 training sessions); session duration in- creased from 40 to 60 min	BDI
9	(47)	Randomized, controlled trial	Adults, fulfilled the ICD- 10 criteria for MDD, aged 18–64 years, *n* = 52; INT: 4362 ± 13.3 y CON: 48.81 ± 11.3 y	INT (*n* = 26) CON (*n* = 26)	(a) INT, 10 min warm-up, an interval- training exercise regimen (upper and lower extremity exercise training) with 3 bouts of 5- min workout with an intensity of 40–59% HRR. After the 5- min workouts, participants exercised at a reduced intensity of 20–39% HRR for 5 min, making together 30 min of aero- bic interval training	3 weeks, 5 ses- sions/week (15 training sessions); each session lasted 40 min	BDI and MADRS
10	(28)	Randomized, controlled trial	Inpatients in the Hannover Medical School, fulfilled DSM-IV criteria for MDD, *n* = 42; INT: 44.2 ± 8.5 y CON: 40.9 ± 11.9 y	INT (*n* = 22) CON (*n* = 20)	(a) INT, exercise training with 25 min workout phase on a bicycle ergometer and 20 min with personal preference (cross-trainer, stepper, arm ergometer, treadmill, recumbent or rowing ergome- ter) with an intensity of 50% of maximum oxygen uptake (b) CON, could take part in the daily activity program of the ward (20 min in the morning)	6 weeks, 3 ses- sions/week (18 training sessions); each session lasted 45 min	BDI-II and MADRS
11	(48)	An open-ran- domized, con- trolled trial	Adults, inpatients with a current antidepressant drug therapy, fulfilled DSM-IV criteria for MDD, *n* = 35; INT: 45.3 ± 13.2 y	INT (*n* = 14) INT (*n* = 11), not included in meta- analysis CON (*n* = 10)	(a) INT, aerobic exercise group; the intervention consisted of 30 min of daily brisk walking or jogging with an exercise intensity of 65–75% of age- predicted maximal heart rate (b) INT, stretching group; not included in meta- analysis c) CON, participants received no intervention	10 days, one ses- sion/ day (10 train- ing sessions); each session lasted 45 min	BDI-II
12	(49)	Randomized, controlled trial	Inpatients, aged 18–60 years, fulfilled DSM-IV criteria for MDD, *n* = 43; mean age 40 years	INT (*n* = 24) CON (*n* = 19)	(a) INT, a program of systematic aerobic exercise consisting of 1-h training with an intensity of 50–70% of maximal work capacity (b) CON, the control group attended occupational therapy while the training group exercised	9 weeks, 3 sessions/week (27 training sessions); each session lasted 60 min	BDI
13	(50)	Randomized, controlled trial	Outpatients, aged 18–60 years, diagnosed for MDD. *n* = 29; INT: 48.68 ± 2.3 year CON: 45.33 ± 3.11 year	INT (*n* = 19) CON (*n* = 10)	(a) INT, 5 walks per week (1 was supervised on a treadmill) with 5 km/h average speed; Participants were asked to perform the remaining 4 walks with the same intensity. All patients were medicated with antidepressants (b) CON, were not assigned to take any exercise and remained taking their usual pharmacological therapy	12 weeks, 5 sessions/week (one was supervised); each walk lasted be- tween 30 and 45 min	HAM-D 17
14	(51)	Randomized, controlled trial	Female smokers, aged 18- 55 years, with moderate to severe depressive symp- toms, *n* = 30; INT: 38.0 ± 11.0 year CON: 37.0 ± 10.0 year	INT (*n* = 15) CON (*n* = 15)	(a) INT, participants exercised on cardiovascular equipment of their choice. Sessions comprised of a 5-min warm-up, 20–30 min of aerobic activity and 5 min cool- down. Exercise was gradually progressed from moderate to vigorous intensity. Participants started with 20 min moderate and 4 min vigorous intensity by adding weekly 2–4 min of vigorous exercise –> by week 12, participants completed 3 sessions with 30 min of vigorous intensity (b) CON, they received health education lecture and films	12 weeks, 3 sessions/week (36 training sessions); each session lasted 30–40 min	PHQ-9
15	(52)	Randomized, controlled clinical trial	Outpatients, aged 18 to 55 years, fulfilled DSM-IV criteria for MDD, *n* = 57; INT: 39.76 ± 11.6 year CON: 37.86 ± 9.85 year	INT (*n* = 29) CON (*n* = 28)	(a) INT, exercise session consisted of continuous and intermittent aerobic activ- ity with an intensity of 60% VO2 max at the beginning. Intensity progressively increased up to 85% of VO2max at the end Furthermore, patients started with the selective serotonin reuptake inhibitor sertraline (50 mg/day) (b) CON, patients only had the selective serotonin reuptake inhibitor sertraline (50 mg/day)	4 weeks, 4 sessions/week (16 training sessions); no general exercise duration	BDI and HAM-D
16	(53)	Randomized, controlled trial	Aged between 65 and 85% years, sedentary, diagnosis of MDD, *n* = 121; INT: 75.0 ± 6.3 y INT: 75.0 ± 6.2 year CON: 75.6 ± 5.6 year	INT (*n* = 37) INT (*n* = 42) CON (*n* = 42)	(a) INT, 10 min warm-up, followed by cycling with an intensity that not exceed 70% of their peak heart rate and a 5–10 min cool-down. Patients reach 50 mg Sertraline within 2 weeks (b) INT, 10 min warm-up, followed by cycling with an intensity that maintain the heart rate within the assigned training range of 60% of peak heart rate. The training scheme increased over the weeks, adapting to possible increases in peak heart rate. Patients reach 50 mg Sertraline within 2 weeks (c) CON, patients reach only 50 mg Sertraline within 2 weeks	24 weeks, 3 sessions/week (72 training sessions); each session lasted 60 min	HRSD
17	(54)	Randomized, controlled trial	Depressed patients aged 19–58 years, *n* = 83; mean age 35.5 years	INT (*n* = 48) CON (*n* = 35)	(a) INT, each session consisted of a warm-up routine and stretching exercises, followed by a running programme. Patients continued to receive the usual psychiatric treatment (supportive psychotherapy) (b) CON, Patients only continued to receive the usual psychiatric treatment as provided (supportive psychotherapy)	12 weeks, 3 super- vised sessions/week; no exercise duration pre- scribed	BDI

### Statistical analysis and bias assessment

The SMDs (with 90% CIs) of the outcome were calculated as a measure of the effectiveness of the treatment and could be either positive or negative. We used the adjusted Hedges' g equation (Equation 1) where m_1i_ is the post- treatment mean of the intervention group and m_2i_ is the post- treatment mean of the control group, divided through the pooled standard deviation s_i_. The adjusted Hedges' g equation was used to take small sample biases into account.

The Cochrane Review Manager Software (Version 5.3) was used to calculate the inverse-variance method according to Deeks and Higgins (55), using the random effects model (56). Several forest plots were generated for the outcome categories general exercise interventions, endurance interventions and neuromuscular interventions. The comparing of weaker (<6 PEDro scale) and stronger (≥6 of PEDro scale) studies was performed in a sensitivity analyses for the risk of bias assessment. To examine a potential publication bias, a funnel plot evaluation was performed.

Based on the recommendations of Cohen (57), the value of SMD was classified according to the following scale: 0–0.19 indicates negligible effect, 0.20–0.49 indicates small effect, 0.50–0.79 indicates moderate effect and ≥0.80 indicates large effect.

Further, we conducted a multivariate meta-regression analysis to examine the effects of the moderator variables on the study effect sizes. Our potential moderator variables were number of exercise sessions, frequency of exercise sessions, exercise intensity or exercise session duration.

$$\begin{array}{l} SMD_{i}=\;\frac{m_{1i}-m_{2i}}{s_{i}}\;\left(1-\;\frac{3}{4N_{i}-9}\right) \end{array}$$

## Results

### Trial flow

We identified 10,831 articles as potentially relevant throughout the search procedure (Figure 1, flow chart). After removing duplicates, the remaining 7,012 articles were screened for irrelevant titles. During this step, 5,237 irrelevant titles were removed and the abstracts of the remaining 1,775 potentially relevant articles were studied. One hundred abstracts did fulfill the inclusion criteria and those 100 full-texts were thoroughly studied for eligibility. Another 73 articles did not meet inclusion criteria due to several reasons (e.g., no exercise intervention, no adequate control group, no MDD- diagnosis, exercise intervention was not supervised, participants with further chronic diseases in addition to depressive disorder included, invalid target outcome, etc.). Finally, 27 intervention trials were included in this meta-analytical review.

### Study characteristics and participants

Across the 27 included studies, 17 intervention trials comprised two study arms with an endurance intervention and with a control condition and 10 intervention trials comprised two study arms with neuromuscular interventions and control conditions. In total, 1'452 depressed participants out of the included 27 trials were used for our meta-analysis with meta-regression. 286 participants received neuromuscular exercise (Table 1, study overview of neuromuscular exercise interventions), 508 participants received endurance exercise (Table 2, study overview of endurance exercise interventions), and 658 participants were allocated to control groups. The mean overall sample size was 53.8 participants per study ranging from 19 (42) to 121 (53) participants. All subjects were over 18 years old and either inpatients or outpatients. The training intervention period ranged from 10 days (48) to 32 weeks [i.e., (36)] with a training frequency ranging from 10 sessions in 10 days (48) to one session per week (31). Training session duration differed from 20 min (31) to 120 min (34). Training intensity was described with the Borg Scale in several studies (33, 44, 45), described in MET (50), described as a percentage of the one repetition maximum (38, 39), as a percentage of maximal heart rate (29, 42, 46, 48, 53), resting heart rate (40, 41, 47), or percentage of max. oxygen uptake (28, 49, 51, 52, 54). Two studies did not describe their exercise intensity (35, 36). Several involved randomized controlled trials with Tai- Chi or Yoga interventions also did not describe a specific exercise intensity thus we assumed that hatha yoga interventions count to low- intensity exercise (37), yogasana yoga interventions count to the low- to moderate intensity group (30, 32) and Tai- Chi interventions (31, 34) were grouped to the moderate intensity group. In studies using progressively increased exercise intensity (51, 52) we used the final exercise intensity during the intervention for the multivariate meta- regression analysis.

### Methodological quality of the included studies

The mean study quality of the endurance intervention trials was 6.2 of the PEDro-scale with a range between 4 and 8 (Table 3). There was one trial (43) that distributed the participants into two age-balanced groups instead of randomization. Blinding of subjects and therapists has not been conducted, which is rather difficult within exercise intervention studies.

**Table 3:** PEDro scores and sum of the included endurance intervention trials.

**Author**	**Eligibility specified**	**Subjects randomly allocated**	**Concealed allocation**	**Similar baseline values**	**Blinding of subjects**	**Blinding of therapist**	**Blinding of assessor**	**Dropout <15%**	**Received treatment as allocated**	**Statistical between- group comparison**	**Point measures and variability provided**	**Sum (2–11)**
(41)	+	+	+	+	–	–	+	+	+	+	+	8
(40)	+	+	+	+	–	–	+	–	+	+	+	7
(42)	+	+	–	+	–	–	–	–	+	+	+	5
(44)	+	+	+	+	–	–	+	–	+	+	+	7
(43)	+	–	–	+	–	–	–	+	+	+	+	5
(45)	+	+	–	+	–	–	–	–	+	+	+	5
(29)	+	+	–	+	–	–	+	–	–	+	+	5
(46)	–	+	+	+	–	–	–	–	+	+	+	6
(47)	+	+	+	+	–	–	+	–	+	+	+	7
(28)	+	+	–	+	–	–	–	+	+	+	+	6
(48)	+	+	–	+	–	–	–	+	+	+	+	6
(49)	+	+	+	+	–	–	–	+	–	+	+	6
(50)	+	+	+	–	–	–	+	+	–	+	+	6
(51)	+	+	+	+	–	–	+	+	+	+	+	8
(52)	+	+	+	+	–	–	+	–	+	+	+	7
(53)	+	+	–	+	–	–	+	+	+	+	+	7
(54)	+	+	–	–	–	–	+	–	–	+	+	4

The mean study quality of the neuromuscular intervention trials was 6.6 with a range between 4 and 8 (Table 4). Thus, overall study quality did not differ between endurance and neuromuscular training. There was also one trial (32) without randomization. Only (30) blinded the therapist.

**Table 4:** PEDro scores and sum of the included neuromuscular intervention trials.

**Author**	**Eligibility Specified**	**Subjects randomly allocated**	**Concealed allocation**	**Similar baseline values**	**Blinding of subjects**	**Blinding of therapist**	**Blinding of assessor**	**Dropout <15%**	**Received treatment as allocated**	**Statistical between– group comparison**	**Point measures and variability provided**	**Sum(2–11)**
(30)	+	+	+	+	–	+	+	–	+	+	+	8
(29)	+	+	–	+	–	–	+	–	–	+	+	5
(31)	+	+	–	+	–	–	–	–	+	+	+	4
(32)	+	–	+	+	–	–	+	+	–	+	+	6
(33)	+	+	–	+	–	–	+	+	+	+	+	7
(34)	+	+	+	+	–	–	+	+	+	+	+	8
(35)	+	+	+	+	–	–	+	+	–	+	+	7
(36)	+	+	–	+	–	–	–	+	+	+	+	6
(37)	+	+	+	+	–	–	+	–	+	+	+	7
(38)	+	+	+	+	–	–	+	+	+	+	+	8
(39)	+	+	+	+	–	–	+	+	–	+	+	7

The overall mean PEDro score including all endurance and neuromuscular intervention trials was 6.41 with a range between 4 and 8 (Tables 3, 4). Twenty out of twenty seven included studies reached the determined cut-off PEDro score of ≥6 and therefore, we can generally evaluate the quality of the included studies as strong enough to be methodological sound.

### Risk of bias assessment

**Figure 2:**
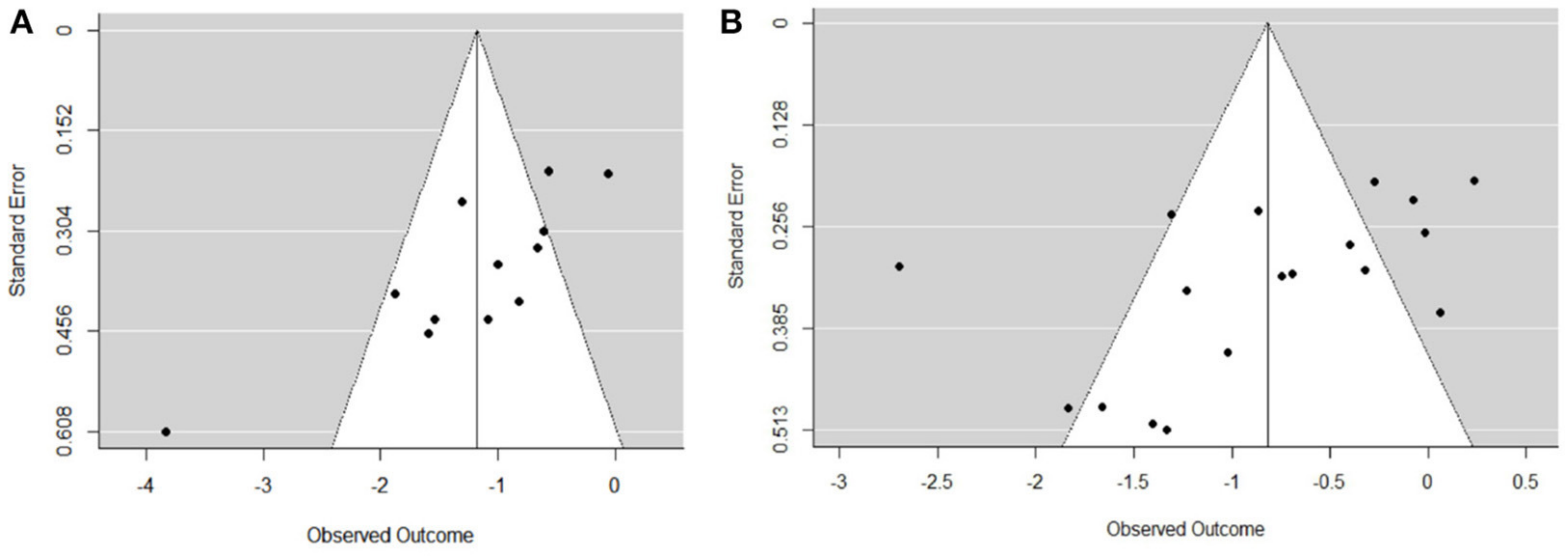
Funnel plots for publication bias in the included neuromuscular intervention studies **(A)** and in the included endurance intervention studies **(B)**.

The funnel plot of the endurance intervention studies (Figure 2) shows an asymmetrical plot and therefore a publication bias cannot be ruled out. It might be plausible that studies with severely depressed patient that did not complete exercise training or refrained from doing it, are not published. Moreover, the control groups underwent other therapies, also pharmacotherapy, this is a considerable bias with lack of stratification. Further analysis revealed that there are several smaller studies (relating to the number of participants), of which results are biased toward larger beneficial effects (29, 42, 46, 48). These studies presented a PEDro score between of five or six, which is below the overall mean score of 6.41. The study of de la Cerda et al. (43) with its effect size of −2.7 attracts also our attention in this funnel plot and is explainable with the low PEDro score and missing randomization.

The funnel plot of neuromuscular intervention studies (Figure 2, plot a) shows a middle asymmetrical funnel shape because smaller studies showing no beneficial effects are missing.

### Effectiveness of an exercise intervention for depression

**Figure 3:**
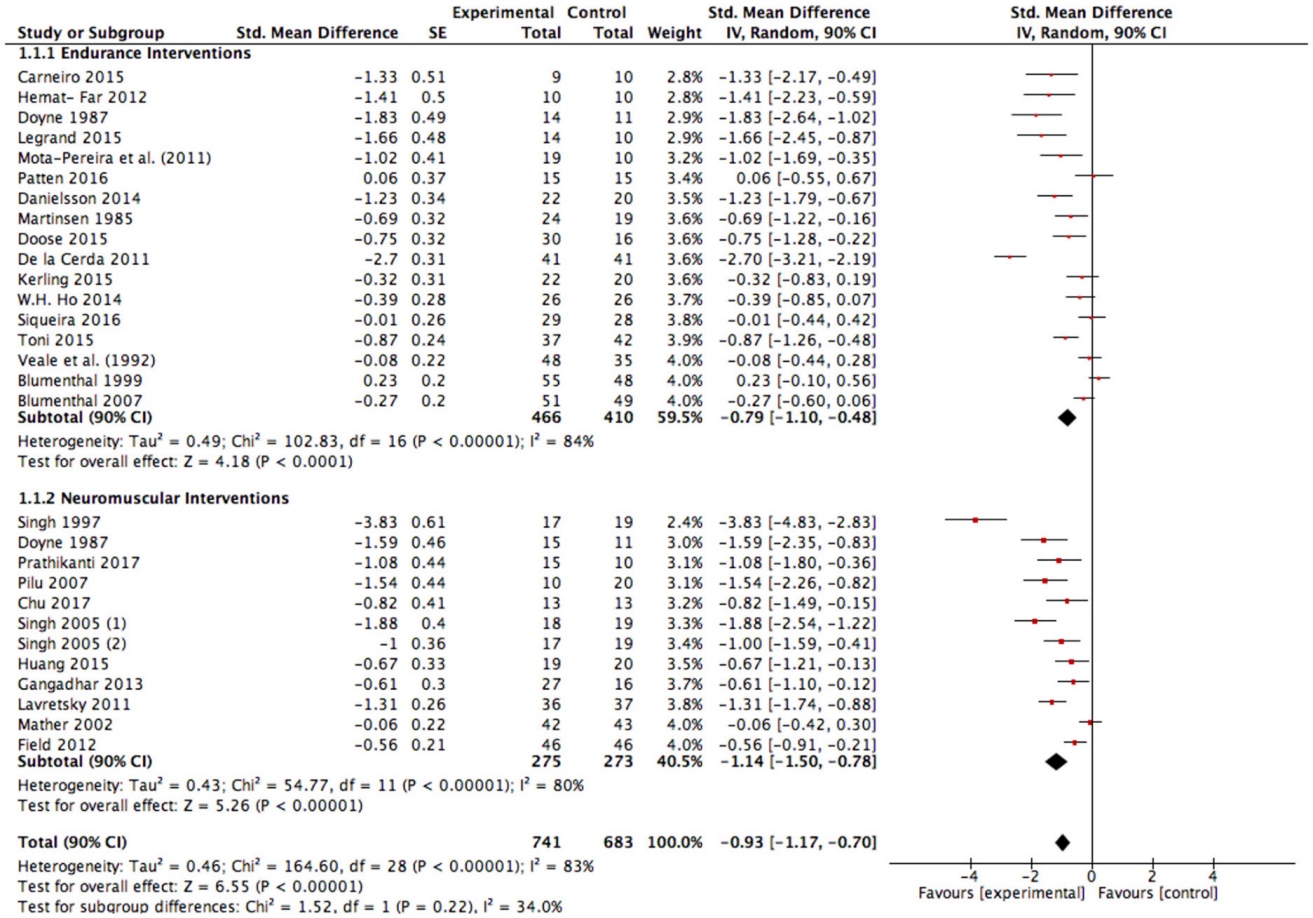
Analysis of depressive symptom outcomes for exercise interventions vs. control groups. SE, standard error; CI, confidence interval; Std., standardized; IV, independent variable.

The results of our meta-analysis with all included studies (Figure 3) show a large effect size of g = −0.93 (90% CI: −1.17 to −0.70); *p* < 0.00001, *I*^2^ = 83%. Heterogeneity can be considered high (*I*^2^ = 83%).

**Figure 4:**
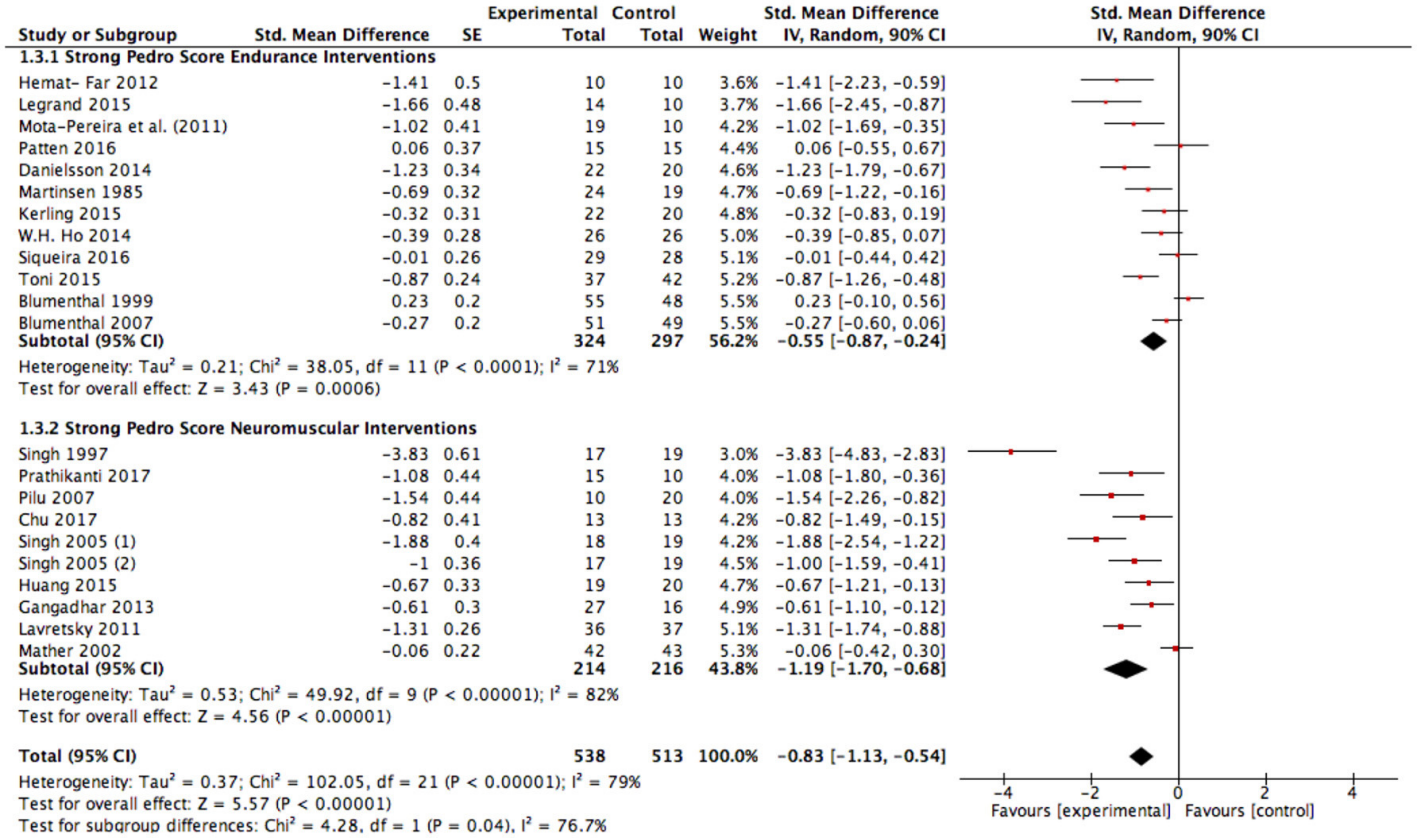
Sensitivity analysis of depressive symptom outcomes for exercise interventions vs. control groups in studies with good methodological quality. SE, standard error; CI, confidence interval, Std., standardized; IV, independent variable.

Further, we conducted a sensitivity analysis due to the methodological weakness of several studies with the assumption that the effect size could be exaggerated in favor of exercise. The pooled data from all included studies with good methodological quality (studies with a PEDro score ≥ 6) still showed a large significant improvement in favor of exercise interventions compared to the control condition [SMD: −0.83 (90% CI: −1.13 to −0.54); *p* < 0.00001, *I*^2^ = 79%; Figure 4]. Heterogeneity can be considered high (*I*^2^ = 79%). Interestingly, neuromuscular training seems to induce significantly higher effects compared to endurance training when considering only strong studies (Figure 4; *p* = 0.04, *I*^2^ = 76.7%).

### Sensitivity analysis of the effectiveness of endurance exercise interventions

**Figure 5:**
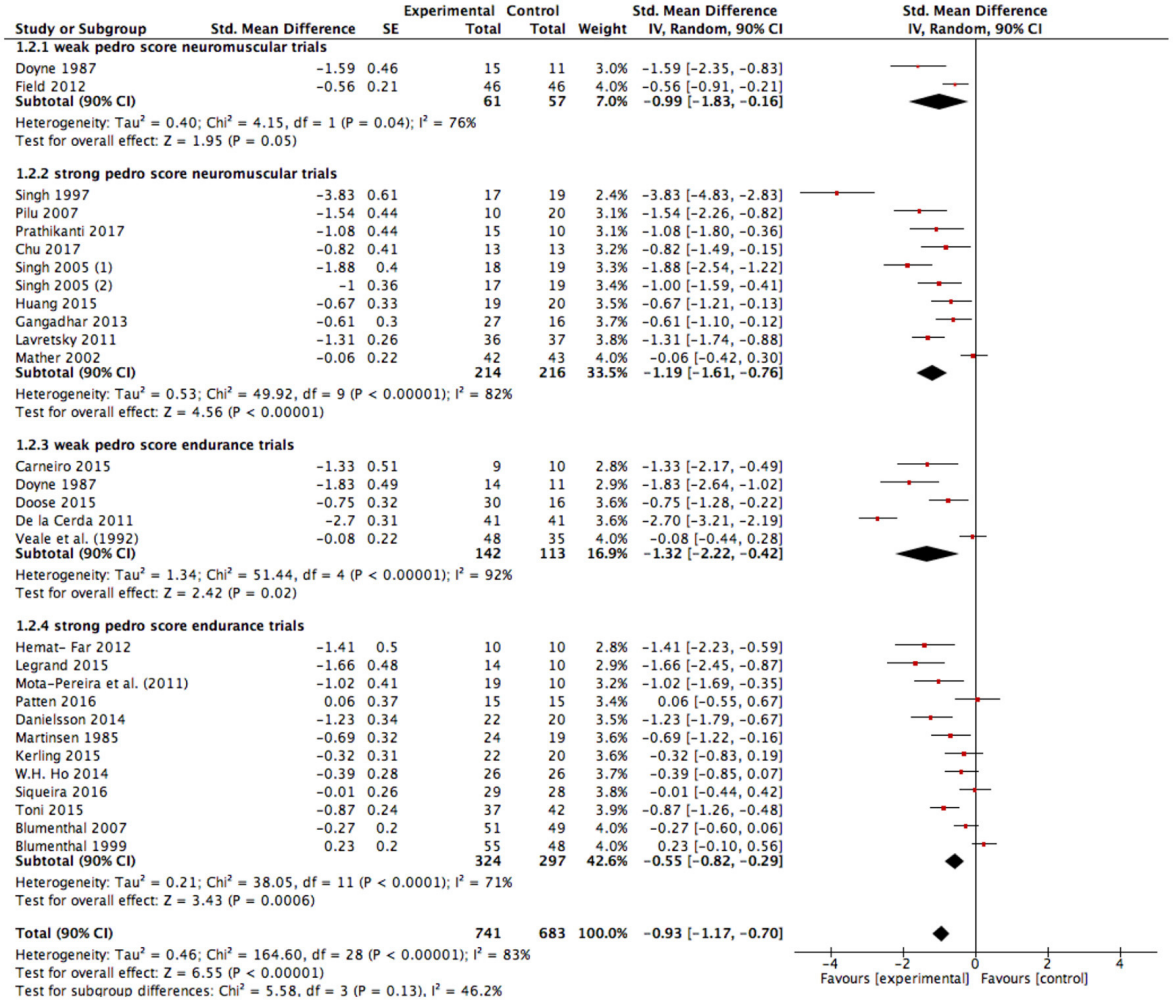
Depressive symptom outcomes for endurance exercise interventions vs. control groups. SE, standard error; CI, confidence interval; Std., standardized; IV, independent variable.

The results of the present meta-analysis show moderate to large effects in favor of endurance exercise interventions compared to the control condition [SMD: −0.79 (90% CI: −1.10 to – 0.48); *p* < 0.00001, *I*^2^ = 84%; Figure 3]. Heterogeneity can be considered high (*I*^2^ = 84%). As explained before and already seen in the funnel plots, risk of bias assessment indicates that weaker studies [PEDro ≤ 5, SMD: −1.32 (90% CI: −2.22 to −0.42), Figure 5] tend to enlarge the effect sizes compared to stronger studies (PEDro ≥ 6, SMD: −0.55 (90% CI: −0.82 to −0.29), Figure 5]. Hence regarding the stronger studies, the effect size is still moderate in favor of endurance exercise interventions compared to control condition.

### Sensitivity analysis of the effectiveness of neuromuscular exercise interventions

Very large effects were found in favor of neuromuscular exercise interventions compared to the control condition [SMD: −1.14 (90% CI: −1.50 to −0.78); *p* < 0.00001, *I*^2^ = 80%; Figure 3]. Heterogeneity can be considered high (*I*^2^ = 80%). Risk of bias assessment revealed that weaker studies [PEDro ≤ 5, SMD: −0.99 (90% CI: −1.83 to −0.16), Figure 5] tend to attenuate the effect sizes compared to stronger studies [PEDro ≥6, SMD: −1.19 (90% CI: −1.61 to −0.76), Figure 5]. Interestingly, the effect tended to be slightly larger in favor of strength/resistance training [SMD: −1.42 (90% CI: −2.21 to −0.64)] compared to Yoga/Tai-Chi approaches [SMD: −1.14 (90% CI: −1.57 to −0.72)]. This finding did not change when adjusting for study quality and strengthened the assumption that neuromuscular training, particularly strength/resistance training can induce superior effects compared to endurance training.

### Meta-regression analysis for effects of endurance training prescriptors

A summary of the results of the multivariate meta-regression analysis in endurance interventions is presented in Table 5. Number of exercise sessions, exercise intensity and frequency of exercise sessions did not moderate the antidepressant effect of endurance exercise interventions. Only exercise duration notably moderated the effect of endurance interventions. An extended exercise duration of 10 min resulted in a significant antidepressant effect size increase of −0.62.

**Table 5:** Multivariate meta-regression analysis results in endurance interventions.

**Moderator**	**Estimate**	**Standard error**	***p***	**95 %-CI**
Intercept	2.46	2.82	0.38	−3.07; 7.99
Duration of sessions	−0.06	0.02	0.01	−0.11; −0.01
Frequency	−0.24	0.18	0.18	−0.60; 0.12
Intensity	0.01	0.02	0.81	−0.04; 0.05
Number of sessions	0.01	0.01	0.50	−0.01; 0.02

### Meta-regression analysis for effects of endurance training prescriptors

A summary of all meta-regression analyses in neuromuscular interventions is presented in Table 6. Number of exercise sessions, frequency of exercise sessions and exercise session duration did not moderate the antidepressant effect of neuromuscular exercise interventions. Only exercise intensity moderated the effect of neuromuscular interventions. An increased exercise intensity of 10% resulted in a significant increased antidepressant effect size of −0.54.

**Table 6:** Multivariate meta-regression analysis results in neuromuscular interventions.

**Moderator**	**Estimate**	**Standard error**	***p***	**95 %-CI**
Intercept	3.133	1.999	0.12	−0.78; 7.05
Duration of sessions	−0.009	0.011	0.44	−0.03; 0.01
Frequency	0.002	0.171	0.99	−0.33; 0.34
Intensity	−0.054	0.028	0.05	−0.11; −0.00
Number of sessions	−0.017	0.02	0.38	−0.06; 0.02

## Discussion

To the best of our knowledge, this is the first meta-analytical review with meta-regression that examined the differential effects of endurance vs. neuromuscular exercise interventions for the treatment of depression taking exercise training prescriptors and study quality into account. A differentiation between neuromuscular and endurance exercise seems beneficial as patients do have different exercise preferences and both exercise modes cause different adaptations on behavioral and molecular level (24). The general effect size of exercise interventions compared to control group conditions was large. Due to methodological issues of some included studies, this effect size can be potentially biased in favor of exercise. A follow-up sensitivity analysis including only methodological sound (PEDro score ≥6) studies led to a reduced, but still large effect size of *g* = −0.83 (see Figure 4). In a second step, the effects of neuromuscular exercise intervention studies as well as endurance exercise intervention studies were investigated and compared to the control condition. Our analyses revealed significant moderate effects for the methodological strong studies for endurance exercise interventions and significant large effects for the methodological strong studies for neuromuscular exercise interventions, respectively. Interestingly, effect sizes significantly differed in favor of neuromuscular exercise training compared to endurance training when only analyzing strong studies. Our multivariate meta-regression analysis for the different exercise training prescriptors revealed potential moderator variables. We found that exercise duration significantly moderates the effect of endurance interventions and exercise intensity moderates notably the effect of neuromuscular interventions.

### Exercise interventions and depressive outcome

Our meta-analysis suggests a large significant overall effect size of *g* = −0.83 for the methodological stronger studies. Previous meta-analyses generally underpin a large effect in favor of exercise based on calculating standardized mean differences. For example, Schuch et al. (20) found an effect size of SMD = 0.98 (95% CI: 0.68–1.28). This effect size was underestimated due to publication bias and was recalculated to 1.11 (95% CI: 0.79–1.43). Schuch et al. (20) noted that the greater effect size in patients diagnosed with MDD results from greater baseline depression scores and a greater potential to reach a larger reduction in depressive symptoms. Compared to our meta-analysis, they did not exclude studies with participants suffering from other chronic illnesses than MDD in their meta- analysis. The meta-analysis of Josefsson et al. (16) showed an effect size of *g* = −0.77 (95% CI: −1.14 to −0.41) and was strengthened by a sensitivity analysis including only studies using intention-to-treat analyses (*g* = −0.70 with 95% CI: −1.03 to −0.38). In line with our findings, further analyses of Josefsson et al. (16) considering only methodological strong studies led to a substantially reduced effect size of *g* = −0.43 (95% CI: −1.06 to 0.21) indicating a moderate but no more a large effect size. They considered methodological quality as being good if a study fulfilled the criteria of an adequate allocation concealment, the use of intention-to-treat analysis and blinded outcome assessment. Since they had only two trials fulfilling these criteria, the result of *g* = −0.43 should be regarded with caution. There was only one available meta-analysis (10) with contrary results on the link between methodological issues and effect sizes. Rethorst et al. (10) found an overall effect size of *g* = −0.80 (95% CI: −0.92 to 0.67) and they noted that studies using intention-to-treat analysis and adequate allocation concealment achieve larger effect sizes. Further meta-analyses revealed only moderate effect sizes, such as Silveira et al. (58) with g = −0.61 (95% CI: −0.88 to −0.33) and (59) with g = −0.68 (95% CI: −0.92 to −0.44) compared to large effect sizes in our meta-analyses. However, their findings might be biased toward an underrated effect in both meta-analyses due to the number of included studies with an inappropriate, active control group [for example (22)] performing low- intensity exercise. Previous studies suggested that also low-intensity exercise has a meaningful effect on depressive symptoms and cognitive variables (60, 61) in patients suffering from depression. Thus, we conclude the inappropriate control group as a reason for the bigger effect sizes in favor of exercise in our meta-analysis compared to earlier meta-analyses. Further meta-analysis and researches should also exclude studies with active control groups, irrespective of the exercise intensity in the control group.

### Comparing of endurance and neuromuscular exercise interventions

Our sensitivity analysis differentiating the endurance and neuromuscular exercise intervention effects resulted in a meaningful difference of effect size when considering strong studies based on PEDro score evaluation. Thus, we conclude that neuromuscular exercise interventions can be more effective than endurance exercise interventions in the treatment of depression. Allocation of patients either to endurance or neuromuscular exercise training programs could be conducted based on individual preferences, emphasizing the potential of strength training. The comparison of our results with earlier meta-analyses is however quite difficult. One available meta-analysis of Silveira et al. (58) is supporting our assumption. They found an effect size of g = −0.96 (95% CI: −1.97 to 0.05) for strength exercise interventions and an effect size of *g* = −0.52 (95% CI: −0.79 to −0.25) for endurance interventions. The effect size is underestimated in both groups because of the inclusion of Krogh et al. (22) showing no effects in favor of any exercise interventions.

In contrast, Rethorst et al. (10) did not support the assumption as they could not find a difference between aerobic and strength exercise. Continuing sensitivity analysis showed that combined aerobic and strength exercise interventions resulted in larger effects. As a consequence, they recommended mixed exercise activities in the treatment of depression. This contrasts with (20) who noted that only aerobic exercise has a large effect on depression, but mixed exercise interventions and resistance exercise interventions have no significant effects. Because of a small number of included studies reporting resistance training in the treatment of depression, their conclusion should be interpreted with caution.

### Training parameters moderate the antidepressant effect of exercise interventions

We found significant moderating effects for the training prescriptors “exercise duration” in the way that an extended exercise duration strengthened the antidepressant effect of endurance exercise interventions. We found an antidepressant effect size increase of −0.62 for exercising 10 min longer. Furthermore, we found significant results for the training prescriptor “exercise intensity” in the way that an increased exercise intensity strengthened the antidepressant effect of neuromuscular exercise interventions. Furthermore, we found an effect size increase of −0.54 in favor of neuromuscular exercise for a 10% increase of exercise intensity. Based on these findings, we cautiously suggest that high intensity neuromuscular exercise can be more effective than low intensity neuromuscular exercise in the treatment of depression. Nevertheless, this conclusion is debatable because of different descriptions of the exercise intensity used in the included neuromuscular intervention trials.

Previous meta-analyses revealed different results in their analyses. Silveira et al. (58) stated that “training parameters such as frequency, intervention period, intensity and duration of training (…) do not exert any influence on the response to treatment” (p. xxx). But they also noted, that the training parameters of most of the included studies were similar concerning to exercise duration, frequency and intensity. However, meta-regression has not been computed in their study.

Another meta-analysis (20) supports our assumption that exercise intensity moderates the antidepressant effect of exercise interventions. They found that moderate to vigorous exercise intensity is more effective than light to moderate exercise intensity. This finding slightly contrasts with (10) who did not find a dose-response relationship between exercise intensity and depression scores in clinically depressed patients. However, they did not differentiate between neuromuscular and endurance exercise. Therefore, a comparison with our results is difficult. Rethorst et al. (10) further found that an exercise duration of 45–49 min results in larger effects than an exercise duration of <45 min or higher than 60 min. These finding supports our conclusion to some extent that longer exercise duration leads to larger effects regarding endurance training.

### Strengths and limitations

The present meta-analytical review was conducted according to the PRISMA statement (25) and along the PICOS approach (27). Our overall sample size of *n* = 1,452 in 27 included trials is presumed to be good and one of the biggest meta-analysis examining the antidepressant effect of exercise interventions. Sensitivity analyses were conducted to differentiate between endurance and neuromuscular exercise interventions as well as between studies with higher and lower quality. The overall mean of the study quality (PEDro score) including all endurance and neuromuscular intervention trials was 6.4 and methodological quality can be assumed to be sound.

The calculated effect sizes were accompanied with a large heterogeneity between the included studies and reasonably narrow confidence intervals. Therefore, our findings provide a comprehensive view of the differential effects of endurance vs. neuromuscular exercise interventions in the treatment of depression.

However, depressive disorders can encompass a plenty of different signs and symptoms which could be also in contrast to each other (DSM-5) such as decreased mood tone, apathy, emotional blunting, hypersomnia, lack of energy, anhedonia, but also irritability, anxiety, hyperphagia, insomnia, and motor activation. A major depressed patient with a melancholic pattern characterized by a marked energy impairment is not likely to benefit from physical exercises more than antidepressant drug administration. Thus, exercise should only be administered as a complementary treatment therapy and not as a substitute for pharmaceutical therapy. In line with this point, a lack of patients' stratification according to severity of symptoms of the underlying studies might provoke a selection bias, as a patient with a high score at HAMD could be less prone to undergo physical exercise in comparison with a patient with less severe depressive symptoms. Moreover, selected studies had to provide adult patients who met criteria for depressive disorder according to DSM IV (validated by SCID), ICD-10, or RDC. On the other hand, BDI-II, GDS, and PHQ-9 are currently used to assess the severity of depressive symptoms, but they are not diagnostic scales. Thus, subgroup analyses on severity of depression and exercise effect was not calculable due to the small resulting sample and used scales. The majority of the included trials show clinical difference between severe, moderate and mild depressions which provoke a lack of stratification which can be clinically relevant too and could be a possible bias. Finally, the control groups have practiced other therapies, also pharmacotherapy, this is a considerable bias with lack of stratification.

Our findings on the influence of different exercise training prescriptors might be biased because of the inclusion of the weaker studies in the analysis. A meta-regression analysis only including the studies with sound methodological quality could lead to different conclusions. Also, the different exercise intensity descriptions in the trials and our transformation to percentage groups to enable adequate comparison may create a limitation to our meta-regression analysis. Further, the blinding of therapist, subject and assessor is impossible in exercise intervention and this may introduce bias. There are also several studies that compared combined exercise and medication treatment to only antidepressant medication treatment. The impact of the antidepressant medication treatment in these studies is unknown and therefore, we do not know how big this potential limitation for our meta-analysis might be.

## Conclusion

Our meta-analysis underpins that exercise training is generally an effective complementary treatment option for depressed patients. Interestingly, neuromuscular exercise interventions can be more effective than endurance exercise interventions when only considering stronger trials. This finding underpins the need of allocating patients to neuromuscular training based on scientific evidence and individual preferences, goals and barriers. To confirm this finding, further randomized controlled trials with clear defined strength training interventions are required. To strengthen our findings regarding the moderation of exercise duration in endurance interventions and exercise intensity in neuromuscular interventions, concurrent training parameters in prospective randomized controlled trials are needed.

Overall, further randomized controlled trials of exercise interventions following the PICOS approach and clearly defined training parameters are required, especially with severely depressed patients in order to state more on the potential of neuromuscular and endurance training in patients that might be less prone to complementary exercise-based treatments. However, strength and endurance training with longer duration and intensities, respectively, should be progressively embedded into treatment regimen of depressed patients.

## Author contributions

LN and LD wrote the whole review of abstract, introduction, material and methods, results, discussion and made tables and figures. LN and LD did study selection and discussed data extraction. LN and EL did methodological quality assessment and statistical analysis. OF and LZ gave comments and advices. AM and MG gave comments and advices to the final version of the manuscript. All authors approved the final version for submission.

### Conflict of interest statement

The authors declare that the research was conducted in the absence of any commercial or financial relationships that could be construed as a potential conflict of interest.
